# Treatment of Diphtheria

**Published:** 1875-11

**Authors:** Lawrence Alexander

**Affiliations:** Yorkville, South Carolina


### TREATMENT OF DIPHTHERIA.

By LAWRENCE ALEXANDER, M.D., of Yorkviele, S. C.

For the benefit of the profession, and other readers of your Journal, I will place before them a treatment on diphtheria, by which the per cent. of casualties has greatly lessened, when compared with practice adopted a few years ago, and still pursued by many physicians throughout the country.

To begin with the preventive treatment, I think very little can be done beyond keeping the health, as near as possible, at a normal standard, guarding, during the prevalence of the disease, against unnecessary exposure to dampness or other atmospheric changes, and keeping, as far as possible, separated from the one already infected, particularly should that one be connected by the ties of blood, which no doubt, as in many other diseases, renders one more susceptible to the influence of the poison.

Unless there is something to counter indicate its use, I invariably commence my treatment with hydg., chlo., mit., combined with ipecac and bicarb. soda, suiting the dose to the age, and repeating every three or four hours. For the use of soda, I am indebted to the American Journal of Medical Science. The modes of action I will not attempt to explain, but its virtue, experience has proved beyond a doubt. This I generally continue for at least twenty-four hours, to be followed, if necessary, by a dose of castor oil, containing a few drops of spirits of turpentine. As soon as practicable, I commence with a saturated solution of sulph. quinia and murated tinct. iron, for here rests the "anchor of hope," as it is all-important to support the system, and build up the red corpuscles of the blood, while the process of eliminating the poison is going on. Of this solution I give every two or three hours, in much larger doses than under ordinary circumstances, continuing throughout the disease, and in reduced doses for some time afterwards.

Commencing with the mercury on first appearance of a deposit upon the mucous membrane of the throat, I apply frequently, say from four to six times a day, with a camel's hair pencil, a solution of carbolic acid, tincture of iodine, and glycerine, varying the strength to suit each case.

In lieu of the above, I have used recently upon several cases, with marked satisfaction, the carbolate of iodine, as prepared by Dr. Kingkendal, of this place. It certainly exercises a most beneficial influence upon the diseased membrane, and as it is a colorless preparation, the objectionable stain of the iodine is avoided. In conjunction with the local applications, I direct the throat to be well gargled every hour, with a solution of chlorate potash, acidulated with carbolic acid, or acid pyrolig, also using this preparation with an atomizer and the mop, no matter what may be the age of the patient, as older persons frequently fail in gargling the throat properly, besides the mop removes loose portions of false membrane, and better exposes the diseased surface to the action of the agents.

In regard to local external applications, I have but little confidence in them. The worry and trouble they give the patient does away with what little efficacy they might possess, and think it only necessary to protect the neck with a piece of flannel in order to regulate the temperature of the parts.

There is generally but little desire for food, but this point should be well carried by the physician, and the patient made to take a sufficient quantity each day, and that selected with an eye to its digestibility.

Of course all cases cannot be treated exactly alike. Some may require a continuation of the mercury almost throughout the disease, with other variations, which precludes the possibility of restricting all cases to the same routine of medication.

It is important to have a moist atmosphere pervade the apartment, and disinfectants freely used. For this purpose the walls and floor should be sprinkled with a solution of carbolic acid, or sol. bromide calcium, at least twice a day.

I think the mistake generally made in the treatment of this scourge of the nursery, is in the too indiscriminate use of the caustic, which adds, by additional irration, to the congestion already existing, and in not paying sufficient attention to the use of disinfectants. Some times, too, those that desire food are either deprived of it, or placed upon an insufficient amount, when it is all-important that the system should be sustained under the wear and tear of the disease; and I am prone to believe that too much reliance is placed upon inexperienced persons in administering remedies, when no disease requires so much the constant attention and observant eye of the physician.

